# Unraveling the Impact of Boron Nitride and Silicon Nitride Nanoparticles on Thermoplastic Polyurethane Fibers and Mats for Advanced Heat Management

**DOI:** 10.1021/acsami.4c06417

**Published:** 2024-07-10

**Authors:** Ahmadreza Moradi, Piotr K. Szewczyk, Aleksandra Roszko, Elzbieta Fornalik-Wajs, Urszula Stachewicz

**Affiliations:** † Faculty of Metals Engineering and Industrial Computer Science, AGH University of Krakow, Krakow 30-059, Poland; ‡ Faculty of Energy and Fuels, Department of Fundamental Research in Energy Engineering, AGH University of Krakow, Krakow 30-059, Poland

**Keywords:** electrospinning, thermal conductive fibers, FIB-SEM tomography, scanning thermal microscopy, nanocomposites, thermoplastic polyurethane, boron nitride, silicon nitride

## Abstract

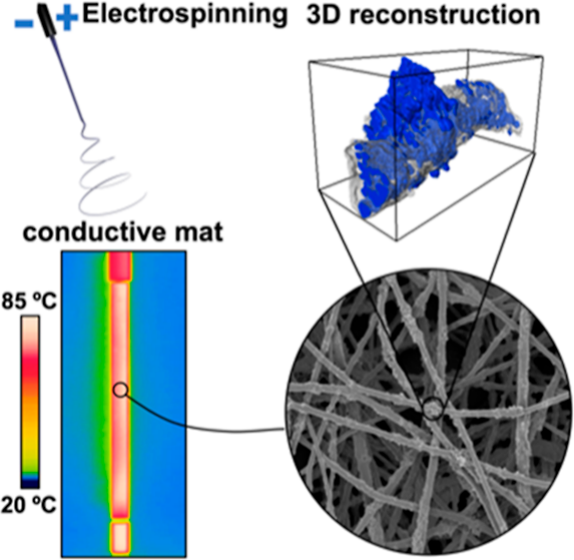

The urgent challenges posed by the energy crisis, alongside the heat dissipation of advanced electronics, have embarked on a rising demand for the development of highly thermally conductive polymer composites. Electrospun composite mats, known for their flexibility, permeability, high concentration and orientational degree of conductive fillers, stand out as one of the prime candidates for addressing this need. This study explores the efficacy of boron nitride (BN) and its potential alternative, silicon nitride (SiN) nanoparticles, in enhancing the thermal performance of the electrospun composite thermoplastic polyurethane (TPU) fibers and mats. The 3D reconstructed models obtained from FIB-SEM imaging provided valuable insights into the morphology of the composite fibers, aiding the interpretation of the measured thermal performance through scanning thermal microscopy for the individual composite fibers and infrared thermography for the composite mats. Notably, we found that TPU–SiN fibers exhibit superior heat conduction compared to TPU–BN fibers, with up to a 6 °C higher surface temperature observed in mats coated on copper pipes. Our results underscore the crucial role of arrangement of nanoparticles and fiber morphology in improving heat conduction in the electrospun composites. Moreover, SiN nanoparticles are introduced as a more suitable filler for heat conduction enhancement of electrospun TPU fibers and mats, suggesting immense potential for smart textiles and thermal management applications.

With the emergence of the 5G era and the rapid development of electronics with higher power density and smaller sizes, the heat generated per unit volume has dramatically increased, which makes it challenging to dissipate heat out of the systems efficiently. On the other hand, problems related to the energy crisis demand immediate actions toward developing energy production and storage systems. In both scenarios, there is a crucial need to develop highly thermally conductive materials.^1−5^ Polymers and composite materials stand at the forefront of this endeavor owing to their versatility and diverse functionalities. Heat spreaders and heat exchangers can be manufactured with superb features like lightweight, corrosion resistance, electrical insulation, compact structure, and low cost if the polymers can be engineered with high thermal conductivity. Working toward this aim, the incorporation of thermally conductive fillers into the polymer matrices has been used as the most efficient and convenient approach.^6−10^ Factors such as the type of filler, its loading percentage, size, and shape highly affect the thermal conductivity of the polymer composites. Moreover, forming a continuous filler network is crucial for achieving high thermal conductivity, which occurs at high filler loading levels. This can lead to poor processability, inferior mechanical properties, and increased cost. Therefore, controlling the spatial arrangement and orientation of fillers are key parameters to design composites with high thermal conductivity at lower filler loadings.^11−15^ Electrospinning offers a versatile and scalable approach for producing composite fibers, meshes, and yarns in a single step.^16−18^ It enables the addition of fillers in high loading percentages and enhances the filler distribution with controllable orientation along the axial direction of polymer fibers.^19,20^ Moreover, the synergy of the applied electrical field and fillers’ organization during electrospinning improves the alignment of the polymer molecule chains, thereby augmenting the intrinsic thermal conductivity and Young’s modulus of the polymer matrix.^21−24^ This enhancement in properties expands their applications in yarns, textiles, aerospace engineering, and biomedical devices.^25−28^ Therefore, understanding the thermal properties of individual micro- and nanofibers is crucial for optimizing material performance and functionality, especially given the unique characteristics that emerge in low-dimensional nanostructures, such as size and temperature dependence of thermal conductivity, and internal phonon boundary and edge scatterings.^29,30^ Scanning thermal microscopy (SThM) with excellent spatial resolution (<50 nm) and thermal sensitivity (<0.01 °C) stands out among methods for characterizing thermal properties, including materials’ thermal conductivity at the micro- and nanoscale. In this technique, a heated nanothermal tip contacts and scans the surface of the samples at room temperature, capturing and processing thermal feedback signals to derive local temperature distribution on the samples.^31−33^

Boron nitride (BN) and silicon nitride (SiN) nanoparticles, possessing high thermal conductivity and mechanical strength, are widely utilized for manufacturing thermally conductive yet electrically insulating composites.^34−37^ According to the literature, BN exhibits a thermal conductivity of up to 200 W m^–1^ K^–1^, generally higher than SiN (up to 180 W m^–1^ K^–1^), and has garnered greater attention for enhancing the thermal conductivity of polymer composites.^13,38,39^ However, there remains a gap in research regarding comparative studies on the role of BN and SiN in the thermal performance of polymer composites. Besides, SiN nanoparticles are commercially accessible in smaller sizes <50 nm in comparison to BN nanoparticles (approximately 150 nm) and, more importantly, with lower prices, highlighting the need for further exploration into the potential of SiN nanoparticles as a competitive alternative. In this research, we aimed for the first time to conduct a comparative assessment on the impact of BN and SiN nanoparticles for enhancing the thermal conductivity of electrospun thermoplastic polyurethane (TPU) fibers and mats. TPU, known for its high tensile strength, excellent chemical and abrasion resistance, good adhesion with various substrates, and outstanding elasticity, presents a promising option for thermal management applications. We produced composite fibers and mats with high concentrations of BN and SiN nanoparticles (30 wt %) and delved into the intricate interplay between the arrangement of nanoparticles, fiber morphology, mechanical properties, and thermal performance of the produced composites. Our findings enabled us to establish a connection between the morphology of the composite fibers and the heat conduction capabilities of the electrospun mats, ultimately identifying the most optimal hybrid system for heat-transfer applications. Figure 1 represents the core concept of this research.

**Figure 1:**
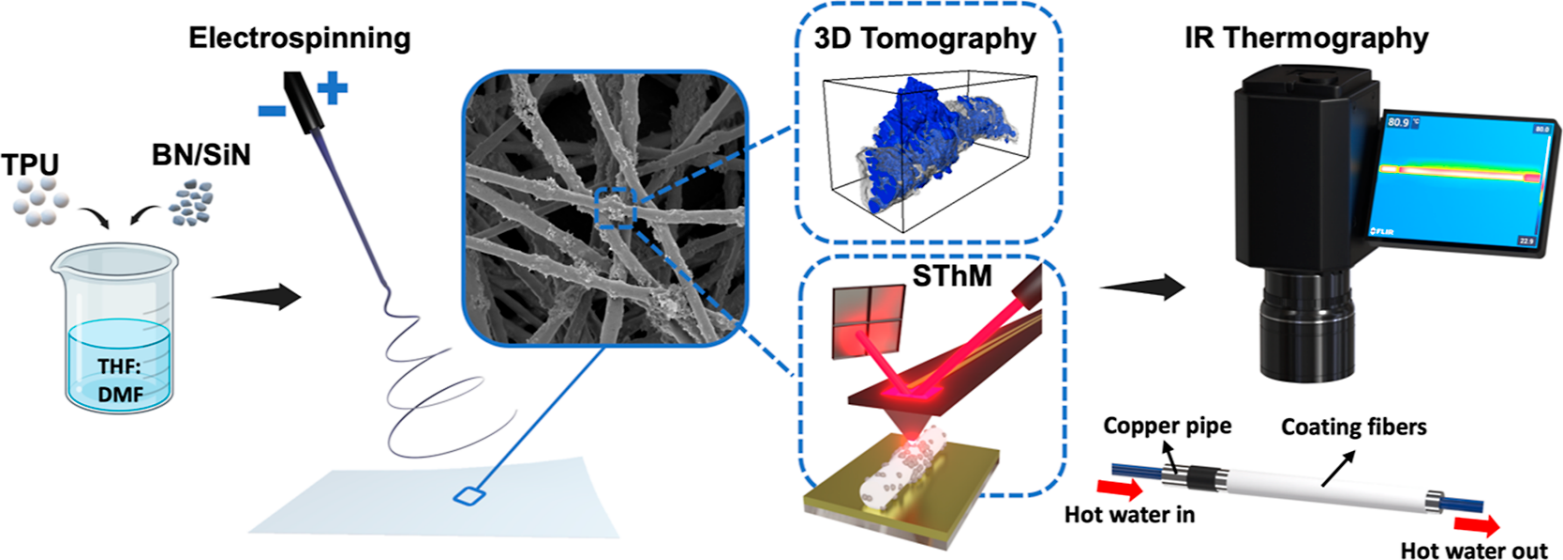
Conceptual schematic of the study on the role of BN and SiN nanoparticles for improving the thermal performance of the electrospun composite TPU fibers and mats.

## Results and Discussion

### Fiber’s Morphology and Density

**Figure 2:**
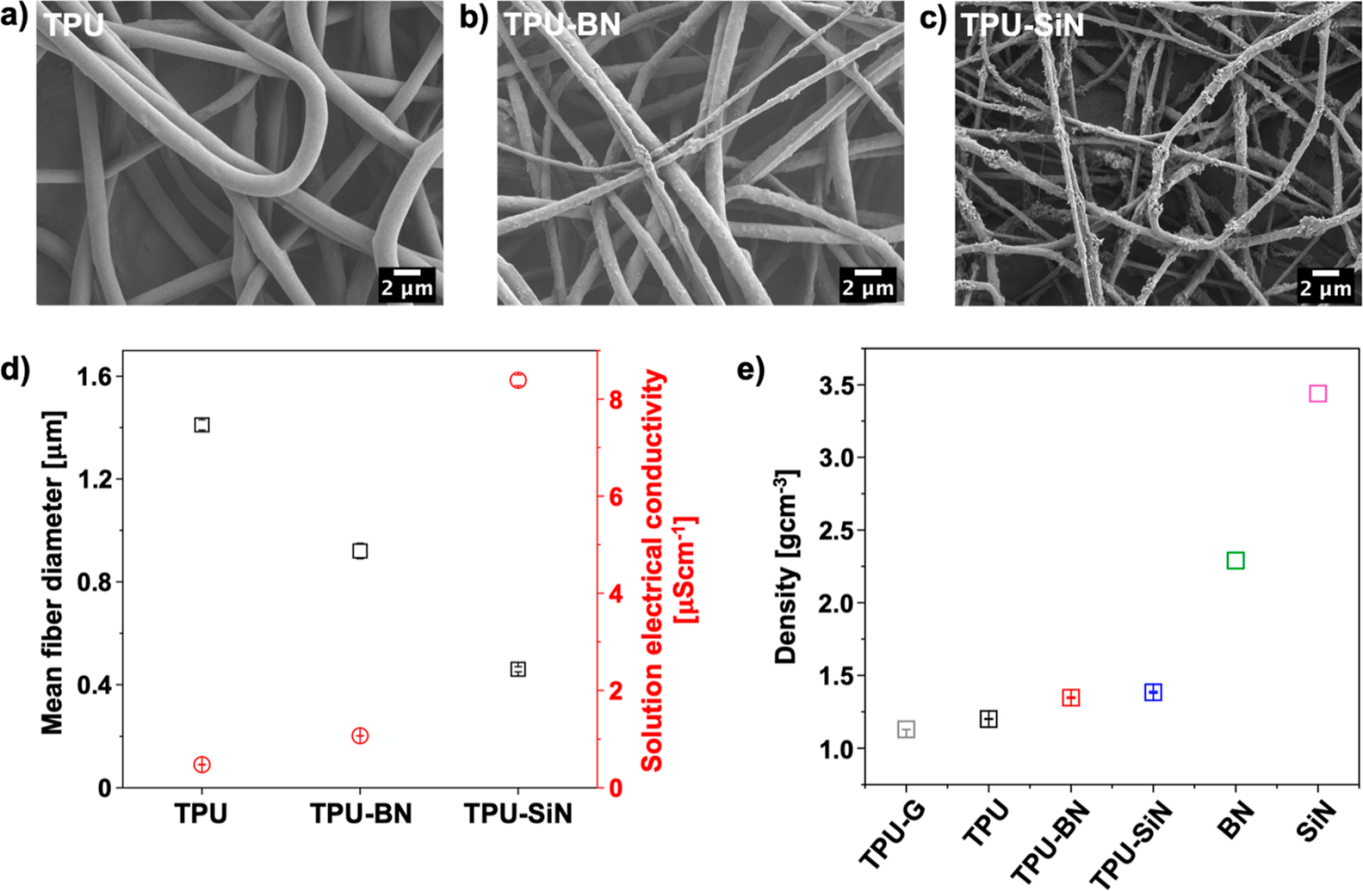
SEM micrographs showing the morphologies of the electrospun (a) TPU, (b) TPU–BN, and (c) TPU–SiN fibers; (d) electrical conductivity of the TPU, TPU–BN, and TPU–SiN solutions before electrospinning and the mean fiber diameter for the 3 types of the electrospun fibers; and (e) measured density of the TPU granules (TPU-G) and the electrospun fibers from pristine TPU and hybrid fibers TPU–BN and TPU–SiN and pure nanoparticles BN and SiN.

The mats based on TPU and TPU mixed with 30 wt % BN and SiN nanoparticles relative to the TPU content were produced using electrospinning. The samples were prepared using similar parameters, but for better processability, the concentration of TPU in the TPU–SiN solution was slightly reduced from 15 to 14 wt % to obtain the optimum viscosity of the solution for electrospinning; the samples are tagged as TPU, TPU–BN, and TPU–SiN. Figure 2a–c presents the morphology of the randomly oriented electrospun TPU and composite fibers. TPU fibers had a smooth surface, which was opposite to TPU–BN and TPU–SiN fibers, where the presence of the BN and SiN nanoparticles formed agglomerates on the surfaces of hybrid fibers. Additionally, the EDS results (Figure S1) indicate that both BN and SiN nanoparticles were present throughout the composite fibers and evenly distributed across the electrospun mats. As illustrated in the diagram in Figure 2d, composite fibers had smaller average fiber diameters. After introducing the nanoparticles, the average fiber diameter drastically reduced from 1.41 ± 0.02 μm for TPU fibers to 0.92 ± 0.03 and 0.46 ± 0.01 μm for TPU–BN and TPU–SiN fibers, respectively. Although the polymer solutions containing nanoparticles had higher viscosity compared to the TPU solution, their higher electrical conductivity increased the surface charge density on the solution jet, consequently enhancing the exerted electrostatic forces in the electrical field. This leads to a higher elongation of the polymer jet, resulting in lower average fiber diameters.^40−42^ Furthermore, the inclusion of the nanoparticles in the fibers increased the density of hybrid fibers. Compared to the pristine TPU fibers, which had a density of 1.2 g cm^–3^, TPU–BN and TPU–SiN fibers showed densities of 1.35 and 1.38 g cm^–3^, respectively, see Figure 2e. This indicates the successful incorporation of the nanoparticles.^43,44^

### Chemical Analysis and Mechanical Properties

**Figure 3:**
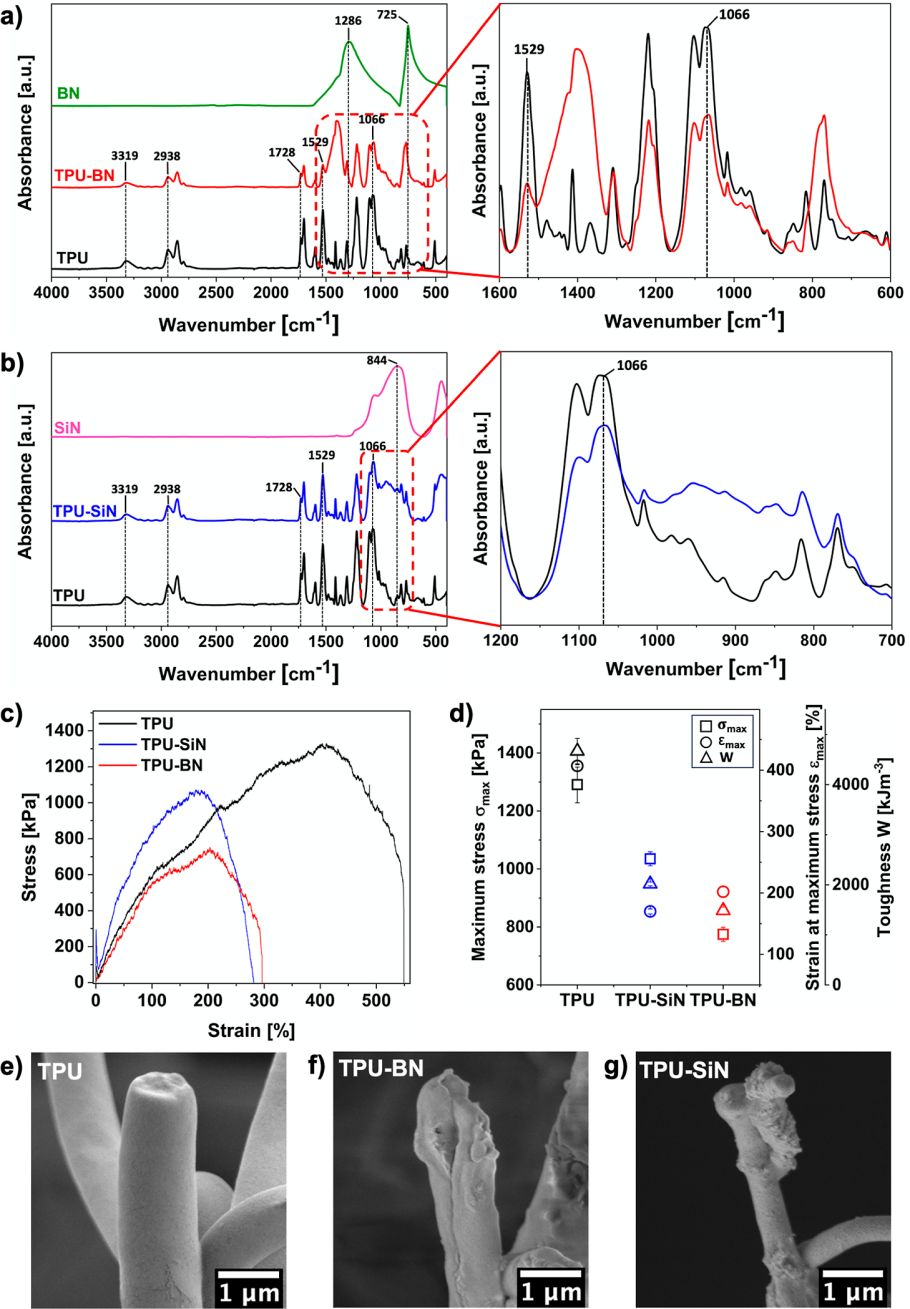
ATR–FTIR spectra of the (a) electrospun TPU and TPU–BN fibers and BN powder with the magnified spectra of the fibers in the range of 600–1600 cm^–1^. (b) ATR–FTIR spectra of the electrospun TPU and TPU–SiN fibers and SiN powder with the magnified spectra of fibers in the range of 700–1200 cm^–1^. The magnified spectra in (a,b) are without the vertical shift of the data. (c) Stress–strain curves of the electrospun TPU, TPU–BN, and TPU–SiN mats, (d) summary of the mechanical properties of the electrospun mat, and (e–g) SEM micrographs of the fractured TPU, TPU–BN, and TPU–SiN fibers after the mechanical test.

The chemical composition of the electrospun mats, as well as BN and SiN nanoparticles, was characterized by ATR–FTIR, see Figure 3a,b. For the TPU mats, the characteristic absorption peak related to the N–H stretching band of urethanes appears at 3319 cm^–1^, and the peak at 2938 cm^–1^ comes from the C–H stretching vibrations. The vibrations of the –H–N–COO– group are represented by the peak at 1728 cm^–1^. Moreover, the peaks at 1529 and 1066 cm^–1^ are attributed to the N–H bending and C–O–C bands, respectively.^45−48^ The spectrum of the BN nanoparticles shows strong absorption peaks at 1286 and 725 cm^–1^, corresponding to the stretching and bending vibration of B–N, respectively.^49−53^ As can be seen in the magnified spectra in Figure 3a, the addition of BN nanoparticles to the TPU mats resulted in peaks with higher intensity in the regions of BN characteristic peaks, confirming the presence of BN nanoparticles in the mats. Similarly, as shown in magnified spectra in Figure 3b, the mats, including SiN nanoparticles, have a higher intensity of peaks in the region 700–1000 cm^–1^, where the SiN nanoparticle spectrum demonstrates the broad peak of Si–N stretch band at 844 cm^–1^.^54,55^

The results from the mechanical testing of randomly oriented fibers are presented in Figure 3c, which shows the representative stress–strain curves of the samples. The TPU mat performed very well during tensile testing with strain at failure (ε_f_) over 500% and a maximum stress (σ_max_) of around 1.3 MPa. However, the addition of high concentrations of BN and SiN nanoparticles lowered the σ_max_ of the TPU–BN and TPU–SiN mats, see Figure 3d; yet, the obtained values still fall within the range of mechanical properties of electrospun mats used in heat-transfer applications.^56−58^ This is due to the nanoparticle agglomerate formation, which induces structural defects within the composite mats and serves as stress concentration points.^54,59,60^ The scanning electron microscopy (SEM) images of the samples after the tensile tests, see Figure 3e–g, confirmed the rupture of the composite fibers close to the agglomerations of nanoparticles. However, compared to the TPU–BN samples, TPU–SiN mats showed higher σ_max_ over 1 MPa. This can be attributed to the smaller nanoparticle size at the same loading percentage of the nanofillers, giving rise to an increased volume fraction of the adsorbed layer of polymers on the nanoparticles’ surface. Since the smaller nanoparticles present in higher numbers and have a higher specific surface area, there are more contact points of a polymer chain with the particles, thereby leading to stronger interfacial interaction.^61^ In addition, as depicted in Figure 2d, TPU–SiN fibers had a much smaller fiber diameter compared to TPU–BN fibers. The reduction of the fiber diameter can increase the elastic modulus of the individual fibers, as the thinner fibers experience higher rates of molecular orientation, resulting in materials with enhanced stiffness.^62−65^ The distribution of nanoparticles within the composite fibers is also another key factor that plays a crucial role in improving the stiffness of the fibers and the mat.^59^ On the other hand, the presence of the agglomerates, which restrict the movement and reorientation of the nanofibers in the direction of the applied load, and the inclusion of the ceramic BN and SiN nanoparticles with low plastic deformation into the polymer fibers are the main reasons for the reduced ductility of the TPU–BN and TPU–SiN samples compared to the TPU mat. As shown in Figure 3c,d and Table S1, both the strain at maximum stress (ε_max_) and ε_f_ of the composite mats were lower in comparison to the TPU sample. However, both the composite mats still demonstrated excellent stretchability and had high strain at failure of over 250%. Furthermore, owing to the reduced tensile strength and ductility of the composite mats, their toughness (W) was lower in comparison to the TPU sample. Notably, the TPU–SiN mat had greater toughness than the TPU–BN mat, see Figure 3d and Table S1.

### SThM and 3D Tomography

**Figure 4:**
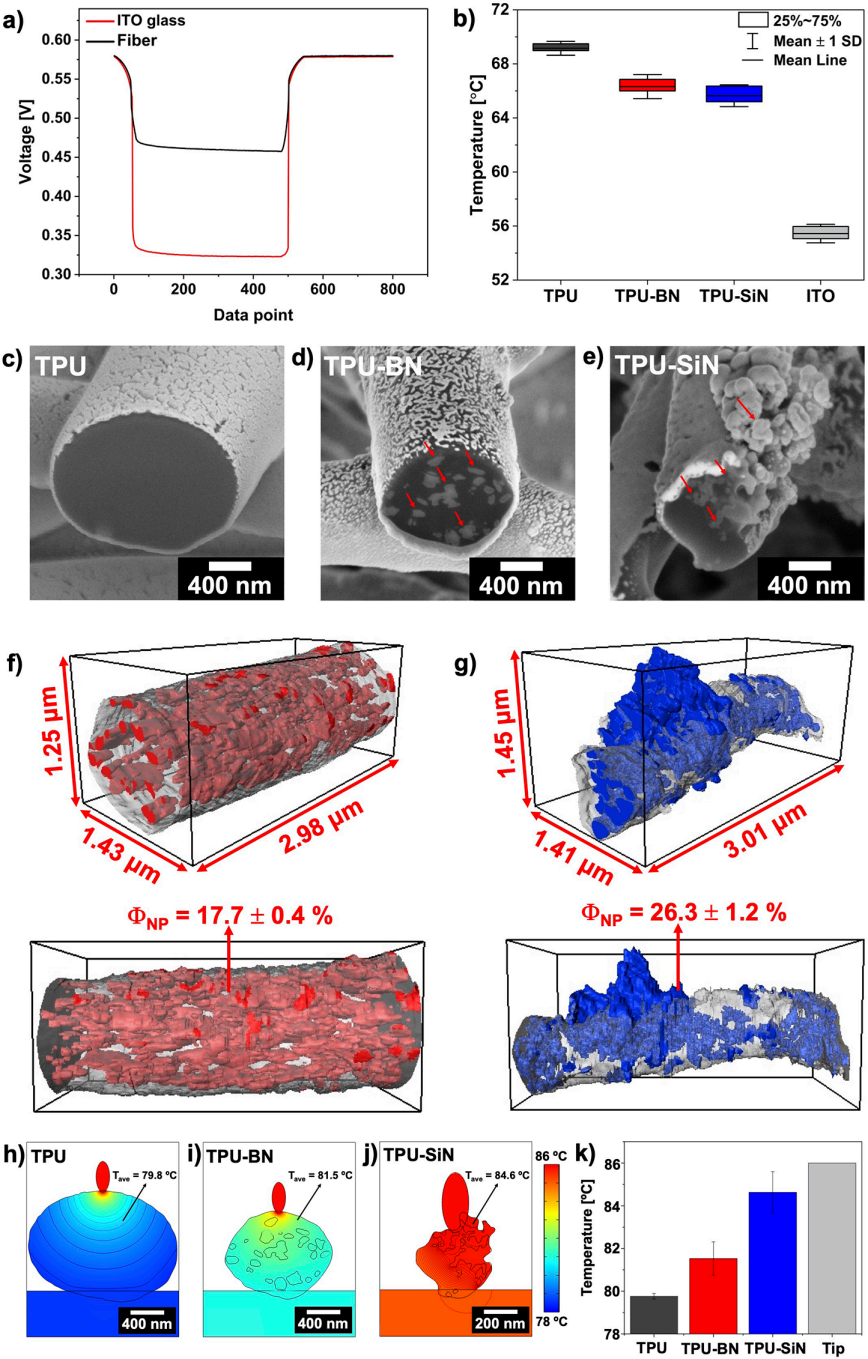
SThM representative of (a) output voltage values obtained on the fiber and on the ITO glass slide as the background during the spectroscopy measurement and (b) average temperature of the tip on the individual fibers and ITO glass. SEM micrographs of the FIB cross-section of the (c) TPU, (d) TPU–BN, and (e) TPU–SiN fibers with BN and SiN nanoparticles indicated by red arrows. (f–g) 3D reconstructions of the TPU–BN and TPU–SiN composite fibers presented in two different perspectives with the BN nanoparticles in red, the SiN nanoparticles in blue, and the TPU matrix in gray. The voxel size was 2.24 × 2.24 × 20 nm in the presented reconstructions. (h–j) Heat-transfer simulation and the corresponding temperature distributions inside the individual TPU, TPU–BN, and TPU–SiN fibers, (k) Column chart representing the average cross-sectional temperature of the fibers.

In this study, SThM method was employed to analyze the thermal performance of the individual electrospun fibers. Figure 4a represents an example of the output voltage values obtained from the SThM tip for one point of spectroscopy measurement on the fiber and one point on the ITO glass as the background substrate. The plateaued region in the middle of each graph corresponds to the cantilever’s tip contact with the fiber or the ITO glass. The lower voltage values for the ITO glass translate to the lower temperature of the tip while it was on the ITO compared to the fiber. This means higher heat transfer and dissipation between the tip and the ITO, as the ITO’s thermal conductivity is higher than that of TPU fiber. Based on the working principles of SThM in thermal conductivity contrast mode, the recorded temperature of the tip is directly correlated with the thermal conductivity of the examined material. The lower temperature means higher thermal conductivity of the material and, consequently, higher heat dissipation of the tip through the material.^31,54,66^

Figure 4b summarizes the result of the SThM measurement on the electrospun fibers and the ITO glass. The average tip’s temperature was lower on the composite fibers compared to the TPU fibers, indicating an enhanced thermal conductivity due to the addition of highly thermally conductive BN and SiN nanoparticles. Furthermore, although the BN nanoparticles generally have higher thermal conductivity in comparison to the SiN nanoparticles,^13,38,39^ TPU–SiN fibers demonstrated a slightly lower average temperature of the tip, around 0.7 °C, which shows their higher thermal conductivity. This difference may be attributed to the morphological variations between the composite fibers. The TPU matrix in TPU–SiN fibers could have higher thermal conductivity compared to the TPU matrix in TPU–BN fiber, which is owing to the smaller fiber diameter and higher structural order of the polymer chains.^23,26^ To gain deeper insights into the morphology of the TPU–BN and TPU–SiN fibers, we employed advanced FIB-SEM imaging, followed by 3D reconstruction of the composite fibers.^67^ This technique provided detailed nanoscale visualization of the nanoparticles’ location within and on the fibers. As shown in Figure 4c–g, despite the relatively uniform distribution of BN nanoparticles in the fiber, there is a lack of continuous interconnections to provide efficient heat-transfer pathways throughout the fiber. On the other hand, SiN nanoparticles were more densely packed and connected in the TPU–SiN fiber, forming a stronger heat conduction network within the composite structure, which could be due to the smaller average size of both the nanoparticles and the fiber diameter. This was further confirmed by calculating the nanoparticle coverage ratio (Φ_NP_) for the 3D reconstructed models of TPU–BN and TPU–SiN fibers. The obtained Φ_NP_ for SiN nanoparticles was 26.3 ± 1.2%, approximately 9% higher than Φ_NP_ for BN nanoparticles, which was equal to 17.7 ± 0.4%. Moreover, contrary to the BN nanoparticles, which were mostly encased in the TPU matrix, SiN nanoparticles were more exposed to the surface of the fibers, see Movies S1 and S2. Therefore, there is a higher likelihood of enhanced heat dissipation of the tip through direct contact with the conductive nanoparticles in the case of TPU–SiN fibers than in TPU–BN samples. However, based on the SEM images, nanoparticles and their agglomerates were randomly distributed on the fibers, and this factor can be involved in the obtained temperature differences between the TPU–SiN and TPU–BN composite fibers. Yet, the spectroscopy measurements were carried out over many randomly placed lines on several fibers to ensure that different parts of the fibers were included in the measurement.

### Simulation of Heat Transfer in the Fiber

To delve deeper into the role of the nanoparticles arrangement and fiber diameter in the thermal performance of the individual composite fibers, we conducted heat transfer simulations for individual fibers. In Figure 4h–j, the temperature distribution in the fibers deposited on the ITO substrate and heated by the SThM’s tip is illustrated. Here, both composite fibers exhibited higher average temperatures compared to the TPU fiber, confirming their enhanced thermal conductivities associated with the presence of the BN and SiN nanoparticles within their structure, see Figure 4k. Furthermore, the TPU–SiN fiber displayed higher average temperature and thermal conductivity compared to the TPU–BN fiber, which is due to its smaller fiber diameter and more connections between the conductive nanoparticles, see Figure 4f–g. The results obtained from simulations corroborated the earlier findings from SThM measurements conducted on the individual fibers. However, in the simulations, the temperature of the tip was kept constant, and the calculated average fiber temperatures, after reaching thermal equilibrium, were used to compare the thermal conductivity of the fibers.

### Thermal Camera Measurement

**Figure 5:**
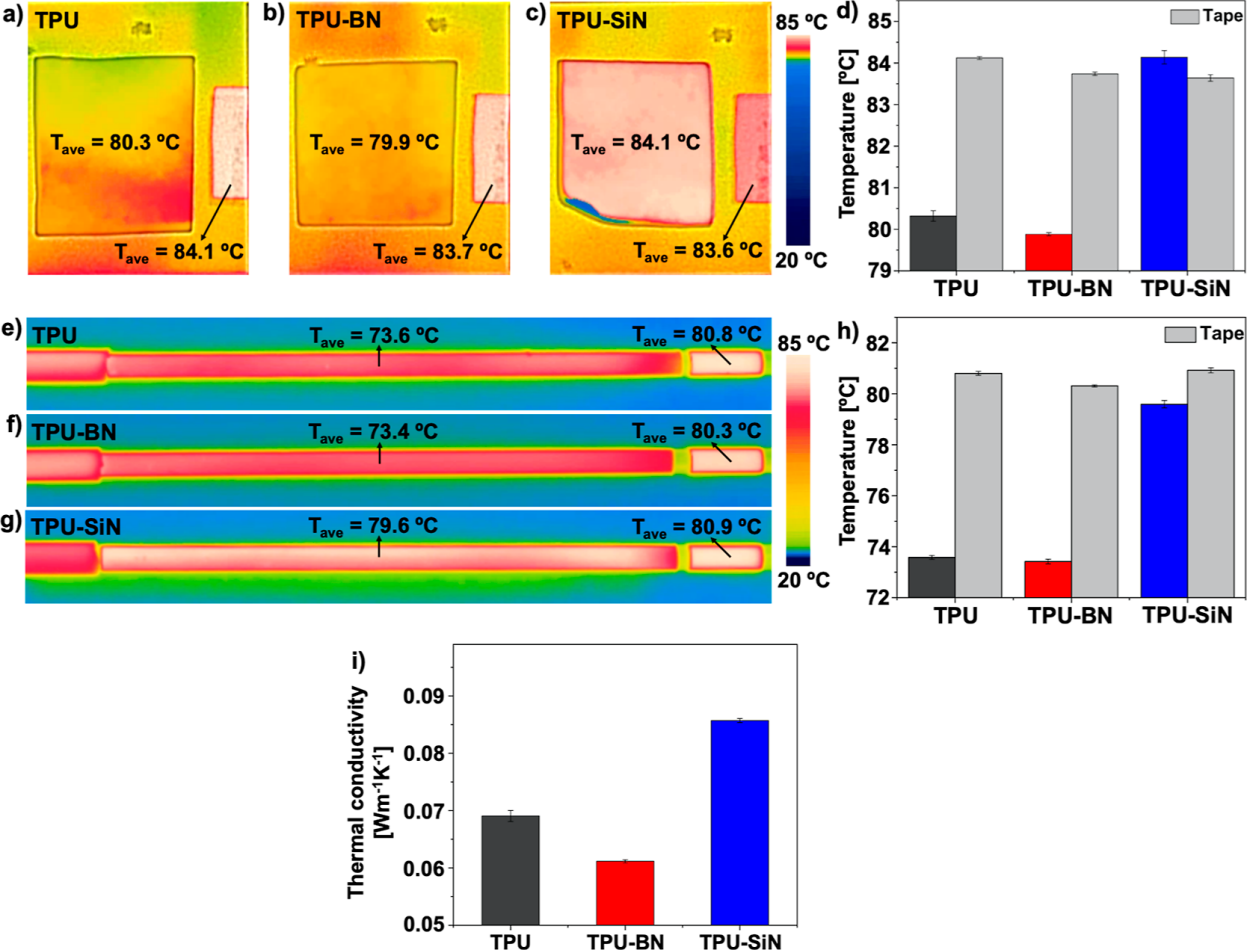
Thermal images of the samples: (a–c) Infrared images of the electrospun TPU, TPU–BN, and TPU–SiN mats on the hot plate set to *T* = 80 °C and (d) column chart indicating the average temperature of the mats and the standard tape. (e–g) Copper pipes covered with the 3 types of electrospun mats while hot water is being passed through the pipes, (h) column chart showing the average surface temperature of the mats and the standard tape on the pipes, and (i) obtained through-plane thermal conductivity values of the multilayered system of the electrospun mats.

To assess further the thermal performance of the electrospun fibers for thermal management, we placed them on a heating plate while recording their surface temperature utilizing the thermal camera setup. In Figure 5a–c, the thermal images of the heated samples after reaching equilibrium are presented. Notably, although TPU–BN mats contained 30 wt % BN nanoparticles, they exhibited similar surface temperature to the TPU mats. On the other hand, TPU–SiN samples had around 4 °C higher temperature compared to TPU and TPU–BN mats, see Figure 5d, which shows that contrary to BN, incorporation of SiN nanoparticles effectively enhanced the thermal conductivity of the electrospun mat. As discussed earlier, the SThM results revealed that both the TPU–BN and TPU–SiN individual fibers had higher thermal conductivity compared to TPU fibers. Nonetheless, regarding the mats, only TPU–SiN samples demonstrated higher surface temperature and heat conduction performance (Figure 5d). This discrepancy is justified by assessing the SEM micrographs of the electrospun fibers, see Figure 2a–c, and FIB-SEM sectioning animations Movies S1 and S2 to compare BN and SiN nanoparticles’ incorporation at the surface of TPU fibers. The contacts between the nanoparticles between fibers in mats form an interconnected network of thermally conductive nanoparticles, resulting in higher thermal conductivity of the porous mats. A similar effect was observed in the literature,^57,68^ where a combination of electrospinning and electrospraying was employed to produce thermal conductive composite mats with interconnecting nanoparticles. The electrosprayed particles placed on the surfaces of the electrospun fibers led to an increased number of heat-transfer pathways and higher thermal conductivity.

Further, our hybrid fibers were evaluated by coating the copper pipes used for transporting hot water. Similar heat dissipation capabilities were observed for TPU and TPU–BN coatings, see Figure 5e–h. Nevertheless, the TPU–SiN sample showed a 6 °C higher surface temperature. As illustrated in Figure S2, the morphology of the fibers coated on the copper pipes resembled the mats placed on the hot plate, which is again the primary reason for the observed thermal performance of the coating mats. Just the TPU–SiN fibers here had slightly lower average fiber diameters compared to their counterparts on the hot plate. Additionally, there was a larger difference between the surface temperatures of the TPU–SiN and TPU coatings on the pipes (6 °C), in contrast to the 4 °C difference between the TPU–SiN and TPU mats placed on the heating plate. This variation occurred despite the TPU–SiN coatings on the pipes having higher thickness than TPU samples, see Table S2. One possible explanation for this observation could be attributed to the lower thermal contact resistance between the coating mats and the pipes since the direct deposition of fibers onto the pipes resulted in better attachment. However, during hot plate measurements, an air gap layer exists between the heating cell and the mats, which acts as a barrier for efficient heat transfer between the heating cell and the fibers.

The obtained results demonstrate the significant role played by the morphology of individual composite fibers, including fiber diameter, nanoparticle organization, and agglomeration in affecting the heat transfer performance of the electrospun mat. In the case of TPU–BN, the addition of higher concentrations of the BN nanoparticles was limited by the viscosity of the solution needed for the electrospinning of fibers. Here, the processability and mechanical performance, together with the overall costs of the end products, is compromised. However, electrospinning proves to be an excellent approach for directly covering and coating the target materials with highly thermally conductive fibers in a single-step manufacturing process with an excellent attachment. This not only diminishes the thermal contact resistance between the materials and the mats but also reduces the need to use an adhesive layer to attach the mats to the intended material. Another advantage of the electrospun mats over commercial materials is their flexibility, allowing them to adapt to various shapes and geometries while maintaining low density. Besides, the high porosity and breathability of the mats are advantageous in covering various surfaces.^69^

To provide a better comparison between the heat conduction capabilities of the produced composite mats in this work with the literature and the commercial materials, we aimed to measure the thermal conductivity of the electrospun mats using the laser flash technique (LFA). LFA has emerged as the predominant method for assessing the thermal conductivity of electrospun composites in thermal management and energy storage applications due to its nondestructive nature, high speed, minimal sample preparation requirements, and the ability to measure both the through- and in-plane thermal conductivities.^70−73^ However, measuring the thermal conductivity of the highly porous electrospun mats in this research posed challenges. It necessitated stacking several layers of the electrospun mats to achieve a sufficient thickness (at least ≈600 μm) for obtaining reliable signals in LFA, which was unattainable with a single layer of the samples having a thickness in the range of 70–120 μm, see Figure S3. Besides, the layering approach was only effective for measuring through-plane thermal conductivities and was not applicable to other types of sample holders, including in-plane holder. Figure 5i represents the through-plane thermal conductivity of the structures consisting of several layers of the electrospun mats. As shown, the TPU–SiN mats exhibited the highest thermal conductivity among the samples. Notably, TPU–BN showed a thermal conductivity even lower than that of TPU mats. This could result from incorporating a higher number of TPU–BN layers to attain a thickness similar to that of the TPU and TPU–SiN stacks, which introduces more air gaps into the structure, inhibiting the efficient heat transfer within the system. Therefore, different thermal resistances between the layers and various numbers and thicknesses of the layers prevent correlation of the obtained thermal conductivity values with the thermal conductivity of the individual layers of the composite mats, hindering not only intracomparison but also comparison with findings from other studies. Furthermore, the obtained thermal conductivity values were notably lower compared to those reported in other research, mainly owing to the presence of air gaps between layers acting as thermal barriers for proper heat transfer between the composites’ layers.^43,47,53,74^

On the other hand, issues such as reaching thermal equilibrium in a short time, laser penetration depth comparable to the film’s thickness, and the need to coat the sample with a conductive layer increase the errors for thermal conductivity measurements of thin films by LFA. Particularly for the electrospun composites, high porosity, up to 90%, and anisotropic thermal conductivity further complicate the thermal conductivity measurements.^75,76^ This underscores the imperative for further research to develop more reliable and accurate methods for measuring the thermal conductivity of the electrospun mats.

## Conclusions

In this research, we show how manufacturing and nanoparticle integration in hybrid fibers can enhance the thermal conductivity of composite systems owning great flexibility and high porosity.

We successfully fabricated composite fibers and mats using electrospinning, wherein SiN and BN nanoparticles were integrated into TPU to enhance their thermal conductivity. Advanced microscopy techniques such as FIB-SEM and the 3D reconstructions revealed that both the SiN and BN were properly distributed in the electrospun fibers. However, BN nanoparticles were embedded mainly within the fibers and isolated from one another, whereas SiN nanoparticles not only formed more compact and interlinked configurations but also were more exposed on the surface of the hybrid fibers. These differences in morphology significantly influenced the thermal conductivity of the individual composite fibers, as measured by SThM. TPU–SiN fibers indicated higher heat conduction capacity than TPU–BN fibers, which was supported by the heat-transfer simulations within individual fibers. Furthermore, the heat transfer capabilities of the composite mats were analyzed by heating them using a hot plate and hot water pipes while monitoring the temperature changes with the IR camera. Interestingly, TPU–BN mats demonstrated similar thermal performance to pristine TPU mats. In contrast, TPU–SiN mats displayed up to 6 °C higher surface temperature, confirming their effective enhancement in thermal conductivity. These results underscore the importance of nanoparticle arrangement and organization, as well as fiber diameter, in improving the heat conduction performance of composite fibers and mats. Additionally, we evaluated the applicability of the LFA for measuring the thermal conductivity of the electrospun samples, revealing its limitations for highly porous systems such as electrospun membranes with low thicknesses in the range of 70–120 μm. Moreover, mats containing SiN nanoparticles exhibited greater maximum stress and toughness compared to those with BN nanoparticles, and both types of composite mats displayed exceptionally high stretchability. The composite TPU–SiN fibers and mats produced in this research offer immense potential for a range of applications, such as thermal management, wearable electronics, thermoregulating textiles, and more.

## Experimental Section

### Sample Preparation—Electrospinning

To produce the electrospun fibers, thermoplastic polyurethane (TPU, 1185 A, BASF, Germany) was dried for 4 h at 30 °C (Drying Oven, POL-ECO Aparatura, Poland) before preparing the solutions. The solutions were obtained by dissolving TPU (15 wt %) in dimethylformamide (DMF) and tetrahydrofuran (THF) in a 1:1 volume ratio by stirring on a heating plate (IKA, Germany) at a speed of 200 rpm for 18 h. For solutions containing BN (particle size <150 nm, density: 2.29 g cm^–3^, Sigma-Aldrich, UK) and (SiN, particle size <50 nm, density: 3.44 g cm^–3^, Sigma-Aldrich, UK), first, the nanoparticles were homogenized in DMF/THF using an ultrasonic bath (Sonorex Bandelin, Germany) for 2 h; later, the TPU granules were added to the suspensions and stirred for 18 h at a speed of 200 rpm. Before the electrospinning, the polymer solutions with nanoparticles underwent an additional 2 h ultrasonication. Here, the concentration of the nanoparticles was optimized to ensure not only that the percolation threshold was surpassed but also that a continuous fiber production process was maintained. The solutions’ electrical conductivity was measured using a conductometer (Mettler Toledo SevenCompact S210, Zurich, Switzerland) supplied with a conductivity probe (InLab 720). The electrospinning was conducted using the equipment (IME Technologies, The Netherlands) with a climate control chamber at a relative humidity (RH) of 40% and a temperature (*T*) of 25 °C.^77^ The other electrospinning parameters were set as applied voltage: 20 kV, flow rate: 0.5 mL h^–1^, and nozzle to collector distance: 20 cm. A 21-gauge stainless steel needle was used as the nozzle.^78^ For the mechanical test, the samples were prepared by electrospinning of solutions for 30 min on laser-cut paper frames with rectangular holes (2 × 1.8 mm) mounted on a cylindrical collector with 10 rpm rotation speed.^79^

### SEM and 3D Tomography

The surface morphology of the electrospun fibers was examined by SEM (Merlin Gemini II, ZEISS, Germany). The samples were coated with an 8 nm layer of Au (sputter coater Q150RS, Quorum Technologies, UK), and the images were obtained at an accelerating voltage: 2.5 kV and working distance: 5.5–5.9 mm using an SE detector. The average diameter (*D*) of the fibers was calculated from 100 randomly selected fibers from SEM images using ImageJ software (v. 1.53d, USA). To analyze the morphology of the fibers after the mechanical test, SEM imaging was carried out at an accelerating voltage: 2.5 kV and working distance: 8.5–9 mm. Elemental mapping using energy-dispersive X-ray spectroscopy (EDS, Bruker, Germany) was carried out to assess the distribution of nanoparticles. The samples were coated with a thin layer of carbon (approximately 15 nm) using a carbon evaporator (K950, Emitech (Quorum Technologies), UK). The mapping was conducted for 300 s at 15 kV, 1 nA, and a working distance of 5.8–6.1 mm, using a backscattered electron detector. For a more comprehensive assessment of the SiN and BN nanoparticle arrangement in the composite fibers, a slice and view procedure was conducted by FIB-SEM (Neon CrossBeam 350, Zeiss, Germany) using Ga^+^ ion beam at 50 pA and 30 kV.^80,81^ The cross-sectional images of the fibers were captured at current: 500 pA, accelerating voltage: 3 kV, and working distance: 5 mm using the in-lens detector. Avizo Fire (v8.1, USA) was used to generate the 3D reconstructions of the composite fibers, following the previous protocols.^82−84^ The nanoparticle coverage ratio (Φ_NP_) was calculated for the composite fibers using the Analyze Particles function in ImageJ software. The Φ_NP_ was calculated from 150 cross-sectional slices generated by the Avizo Fire software for each 3D reconstructed sample.

### FTIR and Gas Pycnometry

The chemical structure of the produced fibers was examined by attenuated total reflectance–Fourier transform infrared spectroscopy (ATR–FTIR, Nicolet iS 5, Thermo Fisher Scientific, USA) using the diamond crystal. The spectra were collected in the 400–4000 cm^–1^ range and averaged over 32 scans at a resolution of 4 cm^–1^. The peak analyzer and normalize functions in Originpro (2021b, USA) were used for baseline subtraction and normalization of the spectra, respectively.

The density of the electrospun fibers and TPU granule was measured using a gas pycnometer (AccuPyc 1330 He, Micromeritics, Norcross, GA, USA). The experiments were conducted with a 1 cm^3^ cylinder cell, and the average density was calculated based on 10 measurements.

### Mechanical Test

The mechanical properties of the electrospun mats were evaluated using a tensile machine (Kammrath & Weiss, Dortmund, Germany) equipped with a 20 N load cell. The measurements were conducted at an extension rate of 25 μm s^–1^, *T* = 21–23 °C, and RH = 50–56%. To calculate stress, the force measured by the machine was divided by the initial cross-section of the mats. The average thickness of the samples was determined by imaging in the *z*-direction on 5 different spots on the samples using a light microscope (Axio Imager M1m, ZEISS, Germany). The Integrate function in Originpro was utilized to determine the average values of toughness (*W*), tensile strength (σ_max_), and strain at maximum stress (ε_max_) from five separate measurements.^85,86^

### Scanning Thermal Microscopy

To analyze the thermal properties of the individual fibers, SThM based on a thermal probe (VTP-200, VertiSense, AppNano, USA) placed on an atomic force microscope (AFM, CoreAFM, Nanosurf, Switzerland) was utilized. A method similar to our previous work was used to prepare the samples and conduct the measurements.^54^ In brief, the samples were prepared by shortly electrospinning on the ITO glass (Ossila, UK) attached to the collector. The temperature on the cantilever’s tip was set to 85.7–85.9 °C by adjusting the laser position. The measurements were conducted in spectroscopy mode over 10 lines on each sample. The time and amplitude for the approach and retraction of the tip were set to 0.5 s and 3 μm, respectively. The tip was paused for 2 s after coming into contact with the sample and after the retraction, and the stop-by force was 75 nN. The output voltages of the tip, when it was paused in contact with the fibers, were used to calculate the average temperature of the tip on the fibers. The same thermal probe was employed for all the measurements, ensuring no variations in thermal transport linked to the probe. All the measurements were carried out under ambient conditions.

### Thermal Camera Measurement

#### Hot Plate

TPU, TPU–BN, and TPU–SiN mats from random fibers were prepared using the same electrospinning deposition time of 4 h to ensure consistent amounts of TPU and nanoparticles across all the three types of samples. The mats were cut into 3 cm × 3 cm squares and heated by a heating plate (TLC plate heater III, CAMAG, Switzerland). The surface temperature of the mats was captured by a thermal camera (FLIR T560, USA). A standard tape (Super 33+, Scotch, USA) with a constant emissivity of 0.96 was also put next to the samples for better control over the heating plate’s temperature. The average surface temperatures of the samples were calculated using the average box feature in FLIR Tools software, see Figure S5a. The measurements were carried out in ambient conditions.

#### Copper Pipe Coating

The TPU, TPU–BN, and TPU–SiN solutions were electrospun as a coating layer on the copper pipes (*L* = 31 cm, *D*_in_ = 4 mm, and *D*_out_ = 6 mm) inserted in the electrospinning equipment as the collector. The electrospinning was carried out for 1 h for all the samples. To cover the pipes with the uniform distribution of the fibers, the nozzle moved horizontally in reciprocating motions with the speed of 20 mm s^–1^ along a 15 cm distance and 3 s pause at both ends. The rotation speed of the pipes was 20 rpm, and the rest of the electrospinning parameters were kept the same. Hot water with a temperature of 85 °C was pumped through the pipes, and the surface temperature of the electrospun coatings was recorded by the thermal camera. The standard tape was attached to the pipes, and its surface temperature was constantly monitored by the thermal camera to avoid fluctuation of water temperature during the measurements of different samples. The average surface temperatures were quantified by utilizing the average line feature in FLIR Tools software, see Figure S5b. The average lines were placed on top of the pipes to minimize any temperature inaccuracies caused by the curvature of the pipes. The measurements were conducted in ambient conditions.

#### Simulation

The heat transfer within the individual TPU, TPU–BN, and TPU–SiN fibers was simulated by COMSOL Multiphysics (version 5.6, COMSOL Inc., Sweden). Owing to the large length-to-diameter ratio of the fibers, it was presumed that the temperature gradient existed only in the radial direction. Consequently, 3D fiber models were simplified into 2D models, reconstructed from fibers’ cross-sectional slices acquired by FIB-SEM tomography. The size of the models was adjusted to the exact sizes of the fibers in SEM images. To reduce the error stemming from the inhomogeneity of the fibers’ morphology, the simulations were performed on models obtained from 3 randomly chosen cross-section images of each sample. The thermal conductivity of the TPU, BN, and SiN nanoparticles was set to 0.32, 200, and 180 W m^–1^ K^–1^, respectively.^38,39^ A heating source with a constant temperature of 86 °C, represented as a platinum ellipse, was utilized to resemble the metal nanorod at the apex of the SThM’s tip in the measurements. The thermal conductivity of the ITO substrate was set to 11 W m^–1^ K^–1^ according to the values reported in the literature.^87^ The external natural convection function in COMSOL was employed to compute the convective heat-transfer coefficient for the fibers and the ITO substrate. The surface emissivity of the fibers was 0.96, and the ambient temperature was set to 23 °C. It was assumed that the heat transfer between the tip and the environment via convection and thermal radiation was negligible. The simulations were conducted at the stationary conditions after reaching the thermal equilibrium.

#### LFA Measurement

The thermal diffusivity of TPU, TPU–BN, and TPU–SiN mats was analyzed utilizing Laser Flash Analysis (LFA 467 LT HyperFlash, NETZSCH-Gerätebau GmbH, Germany). Electrospun mats were laser cut to fit a foil holder of 12.7 mm diameter. The samples were measured as single layers and a stack of multiple layers. The thicknesses of single layers are presented in Table S2; the multilayer samples of TPU, TPU–BN, and TPU–SiN had thicknesses of 0.61, 0.639, and 0.605 mm, respectively. The measurements were conducted at the following conditions: xenon lamp flash voltage 200 V, pulse width 80 μs, and temperature 30 °C. The data acquisition time was set to 10,000 ms; however, only the data up to 4000 ms were taken into account. Data were analyzed using Proteus software (NETZSCH-Gerätebau GmbH, Germany) and approximated by the built-in penetration model. The thermal diffusivity values were based on at least five measurements. The uncertainty of the measured values was less than 1%. Exemplary raw data and their approximated values are presented in Figure S4 for stacks of TPU, TPU–BN, and TPU–SiN samples. The thermal conductivity of the samples was calculated using eq 1
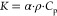
1where α is thermal diffusivity, ρ is the density of the sample, and *C*_p_ is specific heat capacity.

## Supporting Information Available

TPU-BN (400 nm) (MP4)
TPU-SiN (200 nm) (MP4)
SEM micrographs of TPU–BN and TPU–SiN fibers together with their EDS mapping images and corresponding EDS spectra; summary of the mechanical properties of the electrospun TPU, TPU–BN, and TPU–SiN mats; SEM micrographs of TPU, TPU–BN, and TPU–SiN fibers coated on the copper pipes together with their fiber diameter distribution curves; thickness of the electrospun mats; examples of LFA signal for a single layer and multilayer structure of TPU mats; examples of LFA signal for multilayer structure of TPU, TPU–BN, and TPU–SiN mats; and thermal images showing the examples of average boxes and lines used for calculating the average surface temperature of TPU mats and the standard tapes (PDF)

## Abbreviations

BN: boron nitride
SiN: silicon nitride
TPU: thermoplastic polyurethane
FIB-SEM: focused ion beam–scanning electron microscopy
SThM: scanning thermal microscopy
ATR–FTIR: attenuated total reflectance–Fourier transform infrared spectroscopy
ITO: indium tin oxide
LFA: laser flash analysis
DMF: dimethylformamide
THF: tetrahydrofuran
RH: relative humidity
