# Supplementary material for: Unraveling the Impact of Boron Nitride and Silicon Nitride Nanoparticles on Thermoplastic Polyurethane Fibers and Mats for Advanced Heat Management
Source: ACS Appl Mater Interfaces. 2024 Jul 10;16(31):41475–86. doi: 10.1021/acsami.4c06417 (PMC11310906; doi:10.1021/acsami.4c06417)
Supplement: Supplementary file 3 — am4c06417_si_003.pdf [file am4c06417_si_003.pdf]

Supporting Information

Unraveling the Impact of Boron Nitride and Silicon Nitride Nanoparticles on Thermoplastic Polyurethane Fibers and Mats for Advanced Heat Management

*Ahmadreza Moradi[1], Piotr K. Szewczyk[1], Aleksandra Roszko[2], Elzbieta Fornalik-Wajs[2], Urszula Stachewicz[1]**

[1] Faculty of Metals Engineering and Industrial Computer Science, AGH University of Krakow, Krakow 30-059, Poland

[2] Faculty of Energy and Fuels, Department of Fundamental Research in Energy Engineering, AGH University of Krakow, Krakow 30-059, Poland

E-Mail: ustachew@agh.edu.pl

In this supporting information, we provide SEM micrographs of TPU-BN and TPU-SiN fibers together with their EDS mapping images and corresponding EDS spectra (Figure S1), summary of the mechanical properties of the electrospun TPU, TPU-BN, and TPU-SiN mats (Table S1), SEM micrographs of TPU, TPU-BN, and TPU-SiN fibers coated on the copper pipes together with their fiber diameter distribution curves (Figure S2), the thickness of the electrospun mats (Table S2), the examples of LFA signal for a single layer and multi-layer structure of TPU mats (Figure S3),

the examples of LFA signal for multi-layer structure of TPU, TPU-BN, and TPU-SiN mats (Figure S4), and thermal images showing the examples of average boxes and lines used for calculating the average surface temperature of TPU mats and the standard tapes (Figure S5).

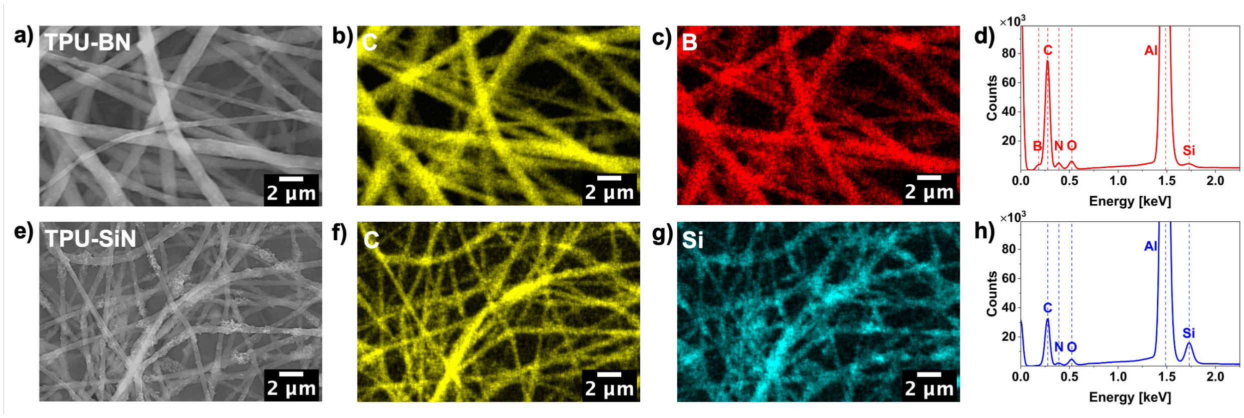

Figure S1. a) SEM micrographs of TPU-BN fibers, and b-d) corresponding EDS mapping images of C and B elements along with the EDS spectrum. e) SEM micrographs of TPU-SiN fibers, and f-h) corresponding EDS mapping images of C and Si elements along with the EDS spectrum.

Table S1. The summary of the mechanical properties of the electrospun mats

	**TPU**	**TPU-SiN**	**TPU-BN**
**Maximum stress σ_{max} [kPa]**	1291 ± 63	1035 ± 24	775 ± 24
**Strain at maximum stress ε_{max} [%]**	407 ± 2	107 ± 4	202 ± 8
**Toughness W [kJm^{-3}]**	4668 ± 254	2017 ± 37	1487 ± 68
**Strain at failure ε_f [%]**	541 ± 4	287 ± 3	286 ± 5

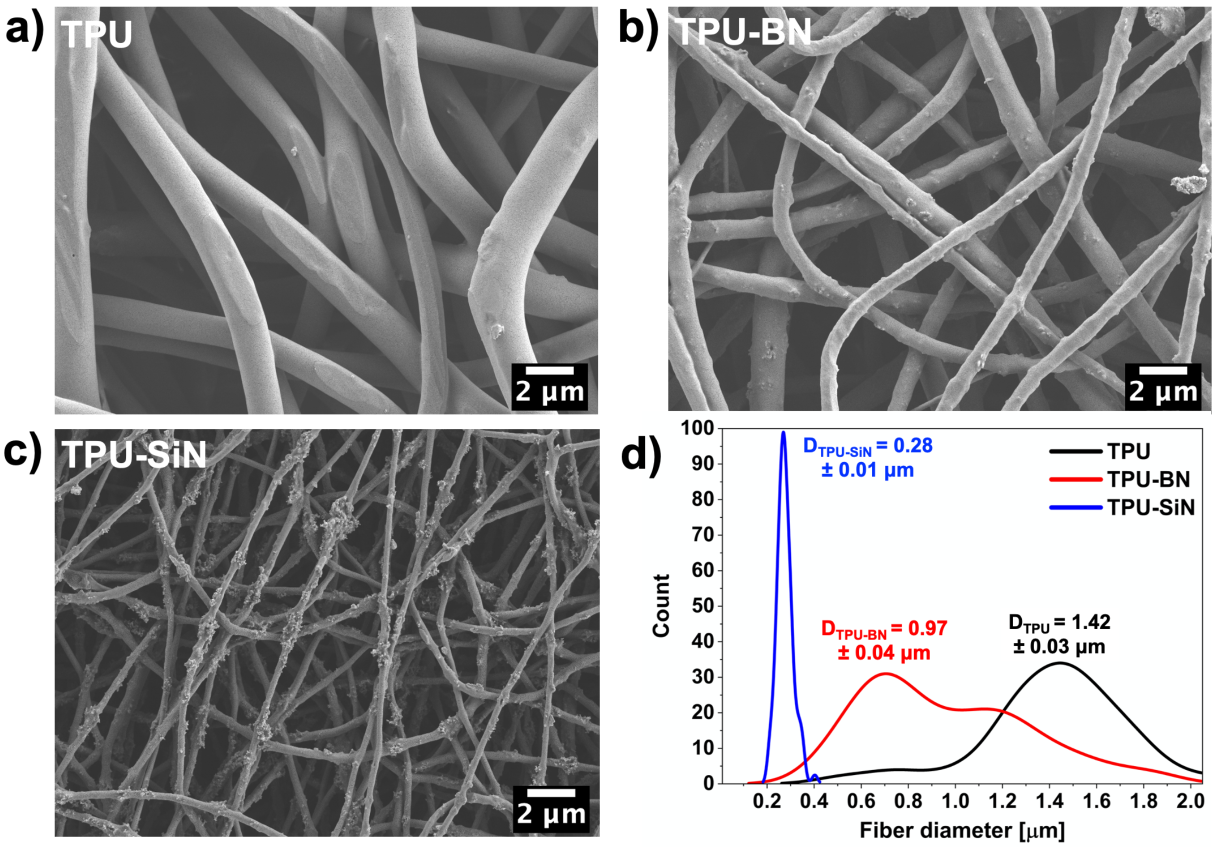

Figure S2. SEM micrographs showing the morphologies of the a) electrospun TPU, b) TPU-BN, and c) TPU-SiN fibers coated on the copper pipes. d) the fiber diameter distribution curves and the average fiber diameters of the electrospun TPU, TPU-BN, and TPU-SiN fibers.

Table S2. The thickness of the electrospun mats on the heating plate and coated on the copper pipes.

	**TPU**	**TPU-SiN**	**TPU-BN**
**The thickness of the electrospun mats on the heating plate [μm]**	122 ± 1	121 ± 3	71 ± 1
**The thickness of the electrospun mats coated on the copper pipes [μm]**	81 ± 3	175 ± 2	116 ± 1

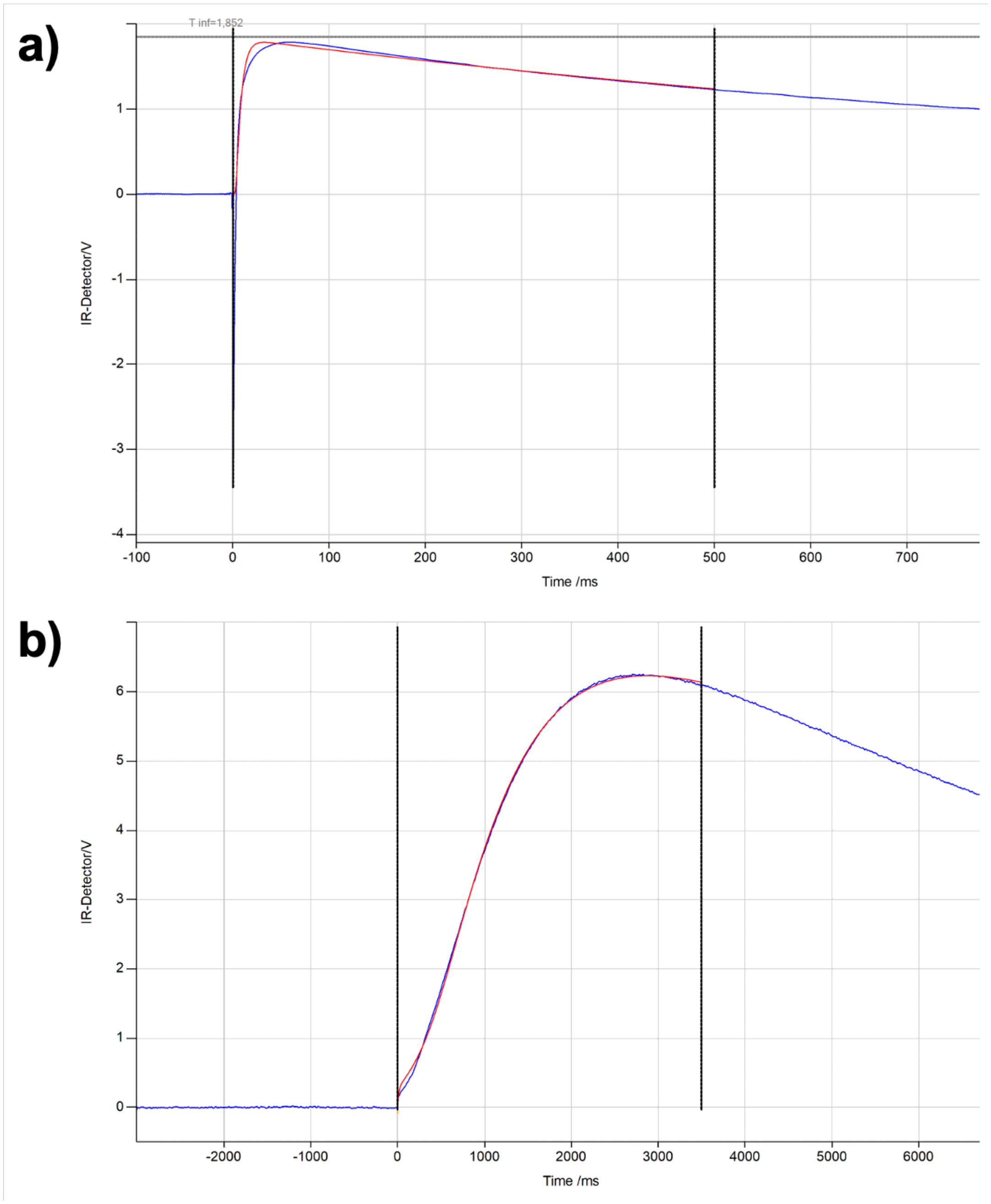

Figure S3. LFA signal examples: a) for a single layer of TPU mat (voltage 200 V, pulse width 20 μs, time duration 5000 ms, temperature 30 °C), and b) for a stack of TPU mats (voltage 200 V, pulse width 80 μs, time duration 10000 ms, temperature 30 °C). The blue line illustrates raw data, while the red line depicts data approximated by the built-in penetration model.

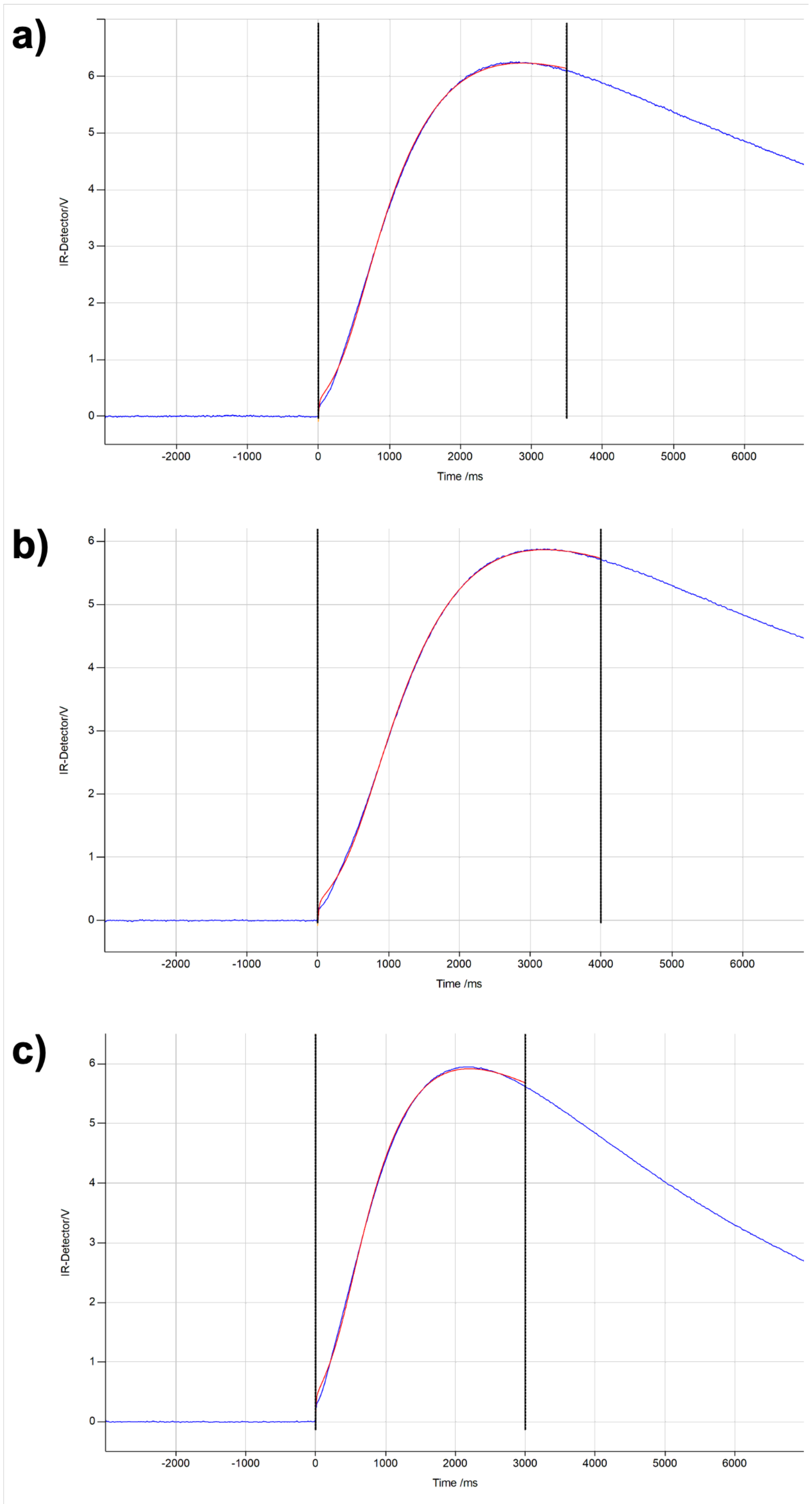

Figure S4. LFA signal examples for a stack of a) TPU, b) TPU-BN and c) TPU-SiN mats (voltage 200 V, pulse width 80 μs, time duration 10000 ms, temperature 30 °C), the blue line represents raw data, while the red line illustrates data approximated by the built-in penetration model.

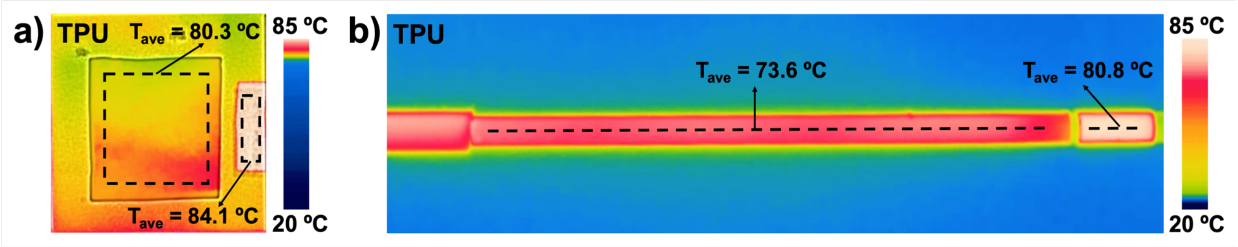

Figure S5. Thermal images showing a) dashed squares representing average boxes used to calculate the average surface temperatures of TPU mats and standard tape on the hot plate, and b) dashed lines representing average lines used to calculate the average surface temperatures of TPU mats and standard tape on the copper pipe.
